# Chromosome-level *de novo* assembly of the pig-tailed macaque genome using linked-read sequencing and HiC proximity scaffolding

**DOI:** 10.1093/gigascience/giaa069

**Published:** 2020-07-10

**Authors:** Morteza Roodgar, Afshin Babveyh, Lan H Nguyen, Wenyu Zhou, Rahul Sinha, Hayan Lee, John B Hanks, Mohan Avula, Lihua Jiang, Ruiqi Jian, Hoyong Lee, Giltae Song, Hassan Chaib, Irv L Weissman, Serafim Batzoglou, Susan Holmes, David G Smith, Joseph L Mankowski, Stefan Prost, Michael P Snyder

**Affiliations:** Department of Genetics, 300 Pasteur Dr, Stanford University, Stanford, CA 94305, USA; Institute for Stem Cell Biology and Regenerative Medicine, 265 Campus Dr., Stanford University, Stanford, CA 94305, USA; Department of Genetics, 300 Pasteur Dr, Stanford University, Stanford, CA 94305, USA; Institute for computational and Mathematical Engineering, Stanford University, Stanford, CA 94305, USA; Department of Genetics, 300 Pasteur Dr, Stanford University, Stanford, CA 94305, USA; Stanford Center for Genomics and Personalized Medicine, Stanford University, 3165 Porter Dr. Palo Alto, CA 94305, USA; Institute for Stem Cell Biology and Regenerative Medicine, 265 Campus Dr., Stanford University, Stanford, CA 94305, USA; Department of Genetics, 300 Pasteur Dr, Stanford University, Stanford, CA 94305, USA; Stanford Research Computing Center, Stanford University, Stanford, CA 94305, USA; Department of Genetics, 300 Pasteur Dr, Stanford University, Stanford, CA 94305, USA; Department of Genetics, 300 Pasteur Dr, Stanford University, Stanford, CA 94305, USA; Department of Genetics, 300 Pasteur Dr, Stanford University, Stanford, CA 94305, USA; School of Computer Science and Engineering, Pusan National University, Busan 46241, South Korea; School of Computer Science and Engineering, Pusan National University, Busan 46241, South Korea; Stanford Center for Genomics and Personalized Medicine, Stanford University, 3165 Porter Dr. Palo Alto, CA 94305, USA; Institute for Stem Cell Biology and Regenerative Medicine, 265 Campus Dr., Stanford University, Stanford, CA 94305, USA; Department of Computer Science, Stanford University, Stanford, CA 94305, USA; Department of Statistics, Stanford University, Stanford, CA 94305, USA; California National Primate Research Center, University of California, Davis, CA 95616, USA; Department of Molecular and Comparative Pathobiology, Johns Hopkins University School of Medicine, Baltimore, MD 21205, USA; LOEWE-Centre for Translational Biodiversity Genomics, Senckenberg 25, 60325 Frankfurt am Main, Germany; South African National Biodiversity Institute, National Zoological Garden, Pretoria, 0184, South Africa; Department of Genetics, 300 Pasteur Dr, Stanford University, Stanford, CA 94305, USA; Stanford Center for Genomics and Personalized Medicine, Stanford University, 3165 Porter Dr. Palo Alto, CA 94305, USA

**Keywords:** chromosome-level assembly, nonhuman primate, pig-tailed macaque, linked-read, HiC

## Abstract

**Background:**

Macaque species share >93% genome homology with humans and develop many disease phenotypes similar to those of humans, making them valuable animal models for the study of human diseases (e.g., HIV and neurodegenerative diseases). However, the quality of genome assembly and annotation for several macaque species lags behind the human genome effort.

**Results:**

To close this gap and enhance functional genomics approaches, we used a combination of *de novo* linked-read assembly and scaffolding using proximity ligation assay (HiC) to assemble the pig-tailed macaque (*Macaca nemestrina*) genome. This combinatorial method yielded large scaffolds at chromosome level with a scaffold N50 of 127.5 Mb; the 23 largest scaffolds covered 90% of the entire genome. This assembly revealed large-scale rearrangements between pig-tailed macaque chromosomes 7, 12, and 13 and human chromosomes 2, 14, and 15. We subsequently annotated the genome using transcriptome and proteomics data from personalized induced pluripotent stem cells derived from the same animal. Reconstruction of the evolutionary tree using whole-genome annotation and orthologous comparisons among 3 macaque species, human, and mouse genomes revealed extensive homology between human and pig-tailed macaques with regards to both pluripotent stem cell genes and innate immune gene pathways. Our results confirm that rhesus and cynomolgus macaques exhibit a closer evolutionary distance to each other than either species exhibits to humans or pig-tailed macaques.

**Conclusions:**

These findings demonstrate that pig-tailed macaques can serve as an excellent animal model for the study of many human diseases particularly with regards to pluripotency and innate immune pathways.

## Introduction

Old World monkeys including macaques share ~93% homology with the human genome and therefore are valuable model organisms for the study of human infectious and genetic diseases [1, 2]. Pig-tailed macaques (*Macaca nemestrina*, NCBI:txid9545) have been widely used as animal models for human infectious diseases, such as human immunodeficiency virus (HIV) and Simian immunodeficiency virus (SIV) [3–8] infections, as well as noninfectious diseases (e.g., neurodegenerative diseases) [9, 10].

Different species of nonhuman primates (NHP) exhibit varying levels of susceptibility to diseases. Studying the variation in disease susceptibility among primate species (including humans) necessitates reliable reference genomes that enable cross-species comparative genomics. However, the qualities of the genome assemblies and annotations for some of the Old World monkeys are inferior to that of the human genome [11]. A major limitation of the current pig-tailed macaque genome assembly (Mnem_1.0) is its reliance on assembly methods that do not yield large continuous genome scaffolds. This hinders comparative genomics studies of disease-related features that might vary, both among NHP species and between these species and humans.

**Figure 1:**
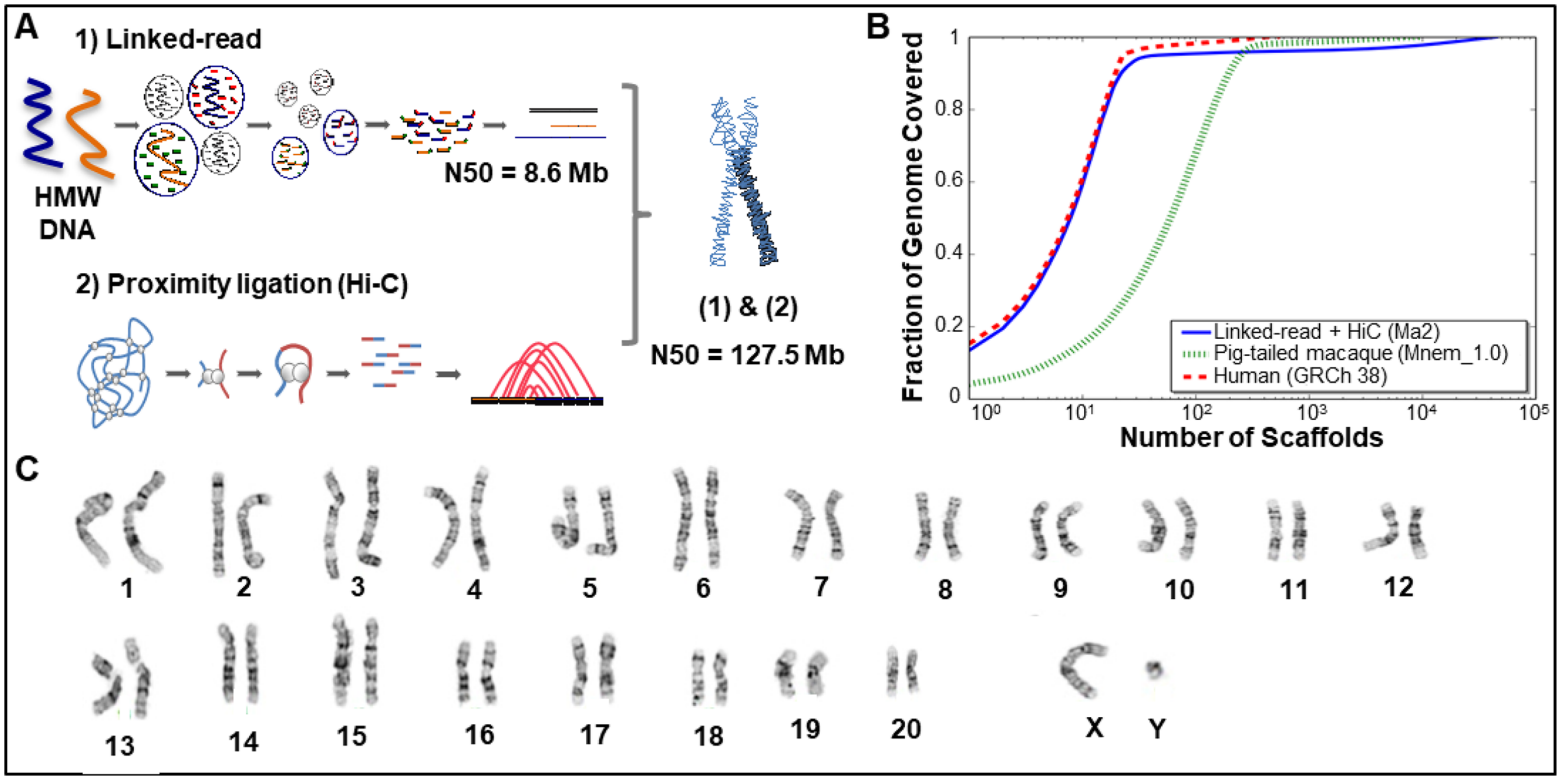
(A) Schematic figure of the methods used for the assembly of the pig-tailed macaque genome (Ma2). (1) The linked-read method resulted in a scaffold N50 of 8.6 Mb. (2) Proximity ligation assay followed by scaffolding using HiC method resulted in a scaffold N50 of 127.5 Mb (almost chromosome level). (B) Comparison of the number of scaffolds (X axis) and the proportion of the genome covered by the assembled scaffolds (Y axis). The 23 largest pig-tailed macaque scaffolds (blue line) cover ~90% of the genome. The red line presents the scaffold sizes of human genome (GRCh38) assembly, and the green line is the current assembly of pig-tailed macaque on NCBI (Mnem_1.0). (C) Karyotype of the pig-tailed macaque chromosomes.

Here we present a high-quality chromosome-level assembly of the pig-tailed macaque (*M. nemestrina*) genome by combining 10X Genomics–derived linked-reads–based *de novo* assembly and scaffolding using proximity ligation (HiC) (Fig. 1A) [12, 13].

This high-quality assembly allowed us to identify large-scale structural variations compared to the human genome. In addition, we annotated the genome using RNA sequencing (RNAseq) and proteomics data from induced pluripotent stem cell (iPSC) lines derived from the peripheral blood mononuclear cells (PBMCs) of the same animal. Using this annotation, we inferred phylogenetic relationships among pig-tailed macaque (*M. nemestrina)*, rhesus macaque (*Macaca mulatta*), cynomolgus macaque (*Macaca fascicularis*), and human (*Homo sapiens*).

## Results

### Chromosome-level assembly of the pig-tailed macaque genome

The presented *de novo* assembly (Ma2) represents a substantial improvement in quality and scaffold size compared to the currently available assembly (Mnem_1.0) and is comparable in quality to the reference human genome (Fig. 1B). Using a combination of linked-reads (10X Genomics Chromium System) and proximity ligation (HiC)-based scaffolding we generated a genome assembly of a total length of 2.92 Gb with a scaffold N50 of 127.5 Mb. The 23 largest assembled scaffolds cover ~90% of the entire pig-tailed macaque genome. We karyotyped the iPSCs from the study animal.

Because the pig-tailed macaque has 20 pairs of autosomes and a pair of sex chromosomes (Fig. 1C) [14], each scaffold likely represents a single chromosome.

With regard to scaffold sizes, the new pig-tailed macaque genome assembly (Ma2) is similar to that of the human genome, which has been continuously improved over the past 20 years since its initial assembly [15]. Using only the linked-reads method we obtained an assembly with scaffold N50 of 8.6 Mb. However, using a combination of linked-reads and proximity ligation, we were able to increase the scaffold N50 to 127.5 Mb. Moreover, we observed significant improvements in reducing the extent of gaps in the assembled scaffolds. To evaluate the quality of our assembly, we ran BUSCO 3.0.2 [16] using the OrthoDB mammalia database. We found 91.9% of complete BUSCO genes in the new pig-tailed macaque (Ma2) assembly, of which 89.0% were single-copy, 2.9% duplicated, 3.9% fragmented, and 4.2% missing (Supplementary Fig. S1A).

### Comparison of the new pig-tailed macaque genome with the human and rhesus macaque genomes reveals both extensive synteny conservation and genome rearrangements

**Figure 2:**
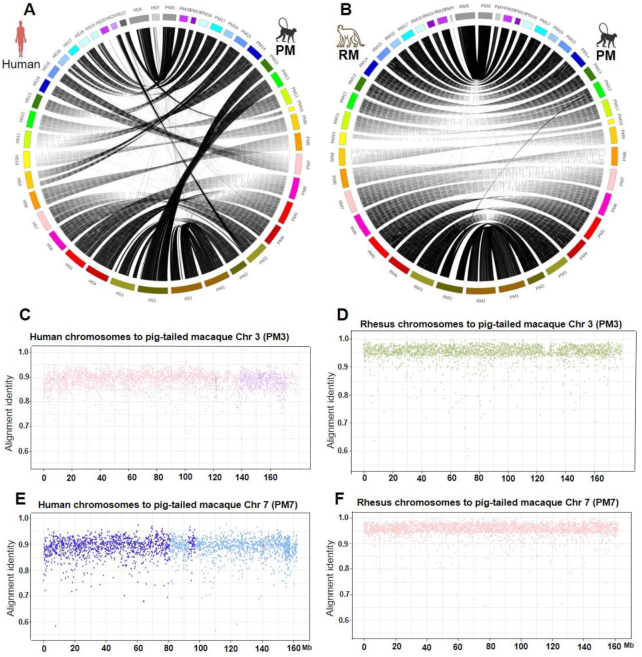
Synteny analysis and structural differences between pig-tailed macaque (PM) chromosomes 1 (PM1), PM chromosome 2 (PM2), through PM chromosomes X (PMX) and Y (PMY) with (A) human chromosomes 1 through Y (HS1–HSY), (B) rhesus macaque (RM) chromosomes. (C) Alignment identity scores between human genome and PM chromosome 3 (PM3), (D) Alignment identity score between RM genome and PM chromosome 3 (PM3), (E) Alignment identity score between human genome and PM chromosome 7 (PM7), (F) Alignment identity score between RM genome and PM chromosome 7 (PM7).

**Figure 3:**
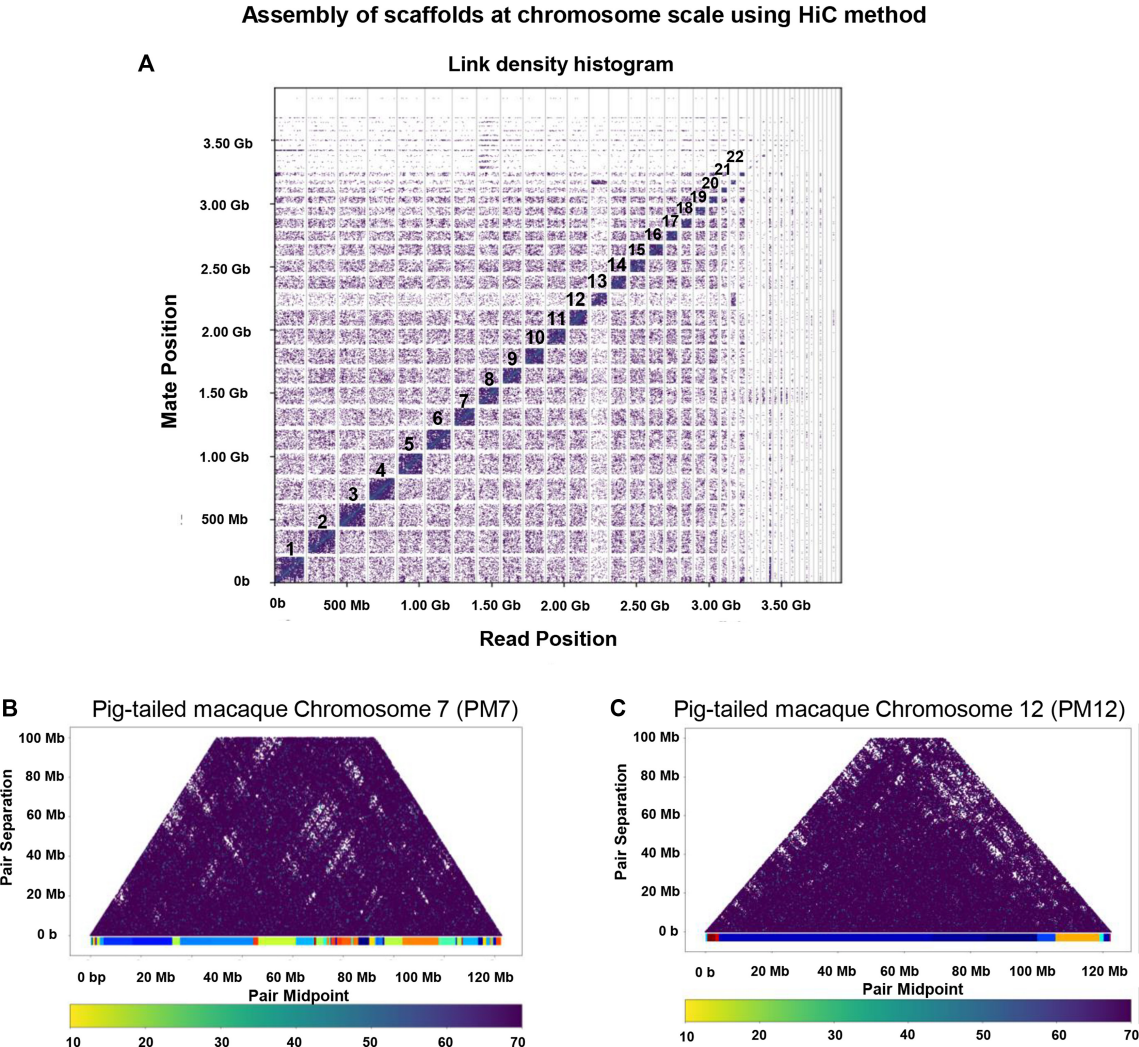
(A) Linked density histogram of the assembled scaffolds of the pig-tailed macaque genome. The numbers mark the largest assembled scaffolds corresponding to the 22 chromosomes. (B, C) Mapping of HiC read pairs on the pig-tailed macaque chromosome 7 (B) and the pig-tailed macaque chromosome 12 (C). Dark purple dots indicate read pairs. As can be seen read pair coverage is even over the chromosomes, indicating that the observed chromosomal changes compared to the human genome are likely genuine and not caused my misassemblies.

Pig-tailed macaques have 20 pairs of autosomes and 1 pair of sex chromosomes (Fig. 1C) [14]. Using the new genome assembly of the pig-tailed macaque, we performed synteny comparison of chromosomes between rhesus and pig-tailed macaque and human and pig-tailed macaque. Synteny analysis among the pig-tailed and rhesus macaque (rheMac 8.0.1) indicated a high level of homology between the two (Fig. 2B–D). Synteny between human and pig-tailed macaque genomes showed large structural rearrangements such as a split of chromosome 7 of the pig-tailed macaque into chromosomes 14 and 15 in the human genome (Fig. 2A, E, and F). In addition, chromosomes 12 and 13 of the pig-tailed macaque both align onto human chromosome 2 (Fig. 2A). We further investigated the read pairing of the proximity ligation libraries for each of these chromosomes to validate the observed large structural rearrangements. The mapping of linked-read HiC data on chromosomes 7, 12, and 13 of pig-tailed macaque supports the accuracy and reliability of the identified rearrangements (Fig. 3A and B).

To rule out biases from repeat homologies, we also conducted synteny analyses comparing the pig-tailed macaque genome with that of human and rhesus macaque after masking repeat elements (Fig. 2A and B). This did not change any of the results discussed above.

### Repeat elements

Our annotation of repeat elements (Table 1) shows similar proportions to those present in rhesus and cynomolgus macaque, and human [1, 17]. The assembly-wide repeat content fraction is 42%, which is also similar to that of other primates including humans [18].

**Table 1:** The statistics of repeats in the pig-tailed macaque genome.

Parameter	No. elements	Length occupied (bp)	Proportion of sequence (%)
SINEs			
Total SINEs	**1,772,888**	**371,166,008**	**12.70**
** Alu/B1**	**1,133,107**	**290,862,183**	**9.95**
** MIRs**	**527,205**	**73,641,539**	**2.52**
LINEs			
Total LINEs	**982,939**	**488,123,096**	**16.70**
** LINE1**	**577,434**	**382,970,388**	**13.10**
** LINE2**	**349,827**	**92,659,483**	**3.17**
** L3/CR1**	**45,394**	**9,932,162**	**0.34**
** RTE**	**9,259**	**2,409,770**	**0.08**
LTR elements			
Total LTR elements	**594,444**	**261,853,336**	**8.96**
**ERVL**	**112,647**	**55,029,397**	**1.88**
**ERVL-MaLRs**	**249,607**	**105,164,349**	**3.60**
** ERV_classI**	**126,831**	**79,524,160**	**2.72**
** ERV_classII**	**80,815**	**15,802,293**	**0.54**
DNA elements			
Total DNA elements	**429,884**	**102,125,714**	**3.49**
** hAT-Charlie**	**220,542**	**44,201,979**	**1.51**
** TcMar-Tigger**	**101,184**	**35,948,175**	**1.23**
**Unclassified**	**54,697**	**5,632,949**	**0.19**
**Total interspersed repeats**		**1,228,901,103**	**42.04**

RTE, non-LTR retrotransposon.

### Pig-tailed macaque (*M. nemestrina*) gene annotation

We annotated the pig-tailed macaque genome using RNAseq and proteomics data from an iPSC line generated from the same animal used for the genome assembly. The iPSC validation for this animal has been conducted as described elsewhere [19]. We also included orthologous proteins of closely related primate species for the annotation. This resulted in the identification of 39,579 transcripts. We then compared orthologous genes across the 3 macaque species and humans (Supplementary Data 1). We identified 26,884 annotated pig-tailed macaque transcripts with no orthologs in either rhesus macaque or human, 8,490 transcripts with orthologs in both rhesus macaques and human, 2,153 transcripts with orthologs in humans only, and 2,052 transcripts with orthologs in rhesus macaque only (Supplementary Fig. S3A).

We next focused on comparing orthologous innate immune system transcripts among macaque species and human to determine whether differences in innate immune response orthologs existed between these species. We used a list of 844 innate immune system genes from the Innate DB database [20]. Interestingly, we observed that several transcripts related to the immune system such as *IL2RA* (NM_0 010 32917.2) and *FAS* (NM_0 010 32933.2) [21–23] had orthologs in the 3 macaque species with no identified orthologs in humans. Among the list of innate immune genes, we also observed *IL31* (NM_0 010 14336.1), *IL36* (NM_01 4440.2, NM_01 9618.3), and *CD101* transcript variant 1 (NM_0 012 56106.2) to have orthologs in human and pig-tailed macaque with no identified orthologs in rhesus or cynomolgus macaques. A list of the genes with orthologs in the 3 macaque species and no identified orthologs in humans is presented in Supplementary Data 4. We also observed that additional genes had annotated transcripts in the 3 macaque species with no identified ortholog in humans; these included *AVPR1B* (NM_0 012 46222.1), the mutations of which correlate with anxiety, depression, and panic disorder in humans [24, 25].

### The use of proteomics data to improve the annotation

We conducted a 2-step search to identify genes corresponding to each protein to improve the annotation of the pig-tailed macaque genome. The first search was against the initial pig-tailed macaque annotation database, created from the RNAseq data; in the second step, unmatched protein spectra were searched against the Human GENCODE19. Our initial Ma2 annotation database identified 9,031 proteins and the Human database identified an extra 4,531 proteins. The total number of identified proteins was 13,562 with false discovery rate (FDR) of 1%. In a further attempt to improve the annotation, we added proteins from the GENCODE 28 database [26] to the input data used for the annotation using Maker 2.31.9 [27]. This resulted in a total of 13,797 proteins used for the annotation. A list of all identified proteins can be found in Supplementary Data 3.

### Pairwise ortholog comparison of macaque species with human

We used POFF (proteinortho5.pl) [28] to infer orthologous relationships between all the annotated genes in the 3 macaque species and humans. From the POFF graph-file output, we extracted the bit scores (a statistical value that represents sequence similarity) for each gene between human and each of the 3 macaque species. We aggregated the bit scores for each gene in the gene sets of interest (the list of innate immune [IM] genes and pluripotent stem cell genes) by computing the mean over all corresponding orthologs for each gene. For genes in the IM and pluripotent stem cell (PSC) gene set, the mean ortholog bit score difference to a corresponding human ortholog is similar for rhesus and cynomolgus macaques, exhibited by data points clustering along the 45° line (Supplementary Fig. S3A and B). However, the mean bit score for several IM or PSC genes is higher for rhesus and cynomolgus compared to pig-tailed macaque, suggesting that the ortholog sequences of rhesus and cynomolgus macaques are more similar to human than those of the pig-tailed macaque. Some genes such as *CXCL12* and *EIF2S2* exhibit more similar sequences between humans and the pig-tailed macaque than between human and either rhesus or cynomolgus macaques (Supplementary Fig. S3A and B).

### Reconstruction of the species phylogenetic tree

In this step, we applied POFF (proteinortho5.pl) analysis to find orthologous sequences among 5 species: the 3 macaque species, human, and mouse. We observed that of the total 124,011 human transcripts with an ortholog from ≥1 other species, 8,286 (6.7%) corresponded to innate immune genes. In contrast, among the total of 4,434 orthologs conserved in all 5 species, 905 (20.4%) belonged to the IM gene set. The overrepresentation of IM sequences in the conserved ortholog set (hypergeometric test *P*-value < 1e−16) suggests that IM genes exhibit higher conservation than other genes. These immune genes include several cytokines, chemokines, and toll-like receptors (*TLRs*).

**Figure 4:**
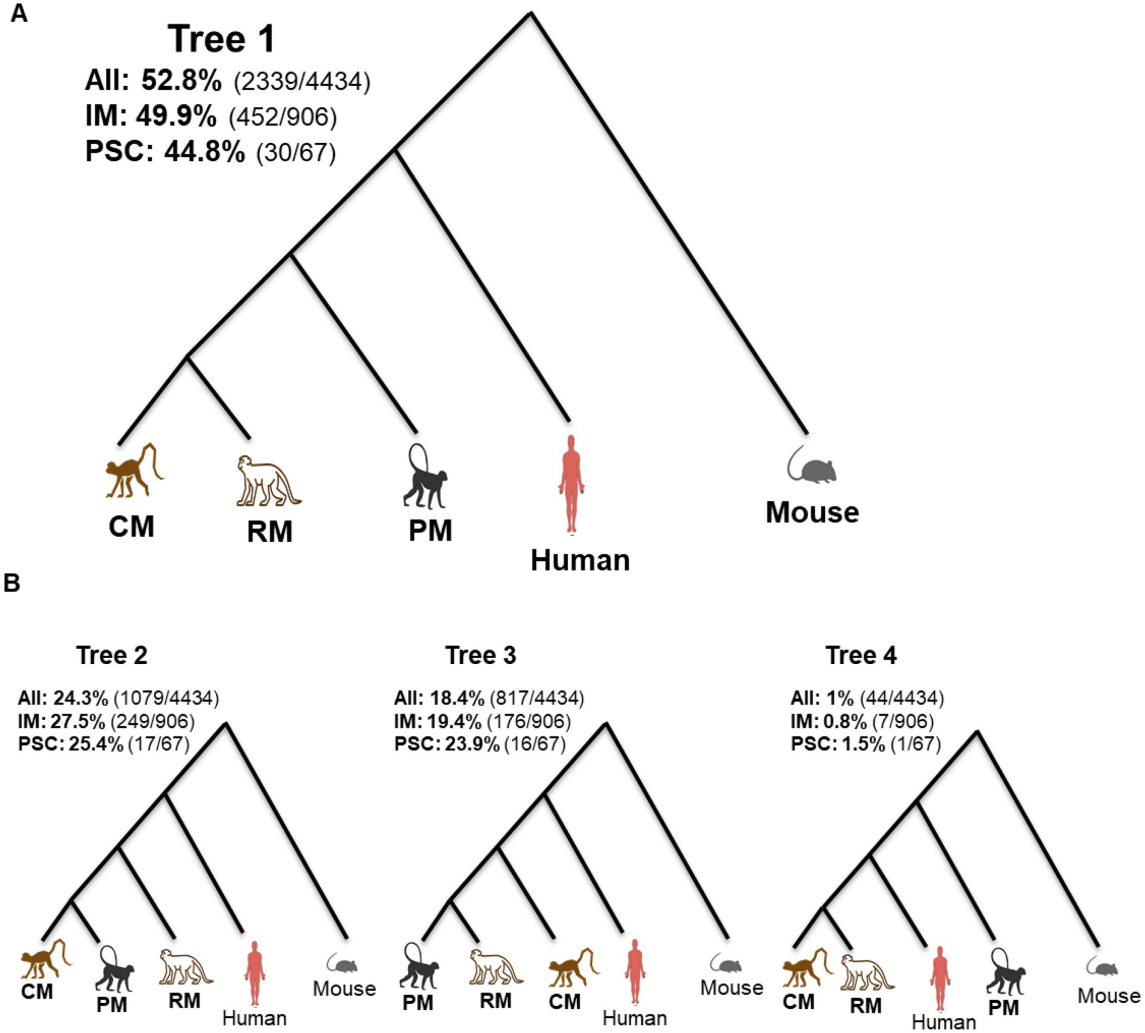
Reconstruction of the phylogenetic relationships among the 3 macaque species and human. (A) The tree topology that is represented by most of the conserved genes among human, pig-tailed macaque (PM), rhesus macaque (RM), cynomolgus macaque (CM), and mouse as an outgroup. Of the 4,434 most conserved orthologs, 52.8% follow the topology of tree 1. Also, 452 (49.9%) orthologs out of 906 most conserved innate immune (IM) genes and 30 (44.8%) of 67 most conserved pluripotent stem cell (PSC) genes follow the topology of tree 1. (B) Trees 2, 3, and 4, each representing different proportions of all of conserved orthologs, IM genes, and PSC genes among the 3 macaque species, human, and mouse.

We further reconstructed phylogenetic relationships among the 3 macaque species and humans using orthologous genes, using mouse as an outgroup. We reconstructed individual maximum likelihood–based gene trees for the 4,434 most conserved orthologs present in all 5 species using RAxML 8.2.10 [29]. Of the 4,434 orthologs identified in all 5 species, 2,339 (52.8%) follow the topology of phylogenetic tree 1 depicted in Fig. 4A. We also investigated the proportion of the IM system genes or PSC genes that follow each of the possible phylogenetic trees in the 3 macaque species, human, and mouse (Fig. 4A and B and Supplementary Fig. S4). Our results indicate that the rhesus and cynomolgus macaques are closely related and exhibit a closer evolutionary distance to each other than either species exhibits to either human or pig-tailed macaque (Fig. 4A). We observed that genes such as *PDX1* and *EIF2S2* follow phylogenetic tree 6 in which the human and the pig-tailed macaque are closely related (Supplementary Fig. S4).

## Discussion

The pig-tailed macaque (*M. nemestrina*) is one of the species of Old World monkeys widely used in biomedical research. However, identification of chromosomal synteny between the genomes of the pig-tailed macaque and humans has not been possible before. This limitation has been mainly due to the low continuity of the current genome assembly, in contrast to the high-quality assembly of the human genome. Recent advances in sequencing and library preparation methods allowed us to generate a genome assembly of chromosome-level quality. In this study, we assembled the pig-tailed macaque (*M. nemestrina*) genome by combining 2 methods, linked-read *de novo* assembly and proximity ligation (HiC)-based scaffolding. This method has resulted in a chromosome-level assembly of the pig-tailed macaque genome. The 23 largest scaffolds cover >90% of the entire pig-tailed macaque genome, a result that is comparable to the human hg38 genome.

Synteny analysis between the human and pig-tailed macaque genomes revealed large-scale chromosomal rearrangements in chromosome 14 and 15 of human with chromosome 7 of the pig-tailed macaque. Moreover, we observed synteny between chromosomes 13 and 14 of the pig-tailed macaque to human chromosome 2. Such large rearrangements were not observed between the pig-tailed and rhesus macaque chromosomes (Fig. 2A and B). The proximity ligation assay data confirmed the authenticity of the observed rearrangements (Fig. 3B).

Next, we reconstructed orthologous relationships among annotated genes of pig-tailed macaques, rhesus macaques, cynomolgus macaques, and human using mouse as an outgroup. Reconstruction of the phylogenetic tree confirms the closer evolutionary distance between rhesus and cynomolgus macaques than between humans and pig-tailed macaques (Fig. 4A). The majority of the genes fit the species tree topology presented in Fig. 4A. A genome-scale estimation of the coalescent-base species tree using ASTRAL 5.6.2 [30] also confirmed the tree topology presented in Fig. 4A [30]. However, some genes do not follow the general consensus species tree (Fig. 4B) (Supplementary Fig. S4).

Because different species of NHP exhibit varying levels of susceptibility to diseases, understanding host responses such as the IM system is crucial for understanding the evolutionary changes that resulted in humans being susceptible to certain diseases. High-quality assemblies and annotations of NHP are essential for such cross-species comparisons. In this study we identified orthologs of some important IM response genes present in pig-tailed macaques and humans but absent in rhesus or cynomolgus macaques, some of which might play roles in disease susceptibility variation. We investigated the evolutionary tree topology for innate immune genes, because of the significant role of innate immune genes in diseases with similar pathophysiology in macaques and humans (e.g., SIV, HIV, and tuberculosis). However, the majority of the IM genes also follow the tree topology of the general consensus tree (Fig. 4A). The comparison of the IM system orthologs is important to understand the variation in disease susceptibility among macaque species and humans. Comprehensive annotations of the 3 macaque species genomes are required for such comparative genomic studies.

iPSCs that can be derived from somatic cells (e.g., peripheral blood cells) have the potential to differentiate into various tissues. Here we used iPSCs of the pig-tailed macaque, generated from the PBMCs of the study animal, for the genome annotation [19]. Differentiation of iPSCs to different lineages in future studies could be a tool for screening the functional roles of different genes and repeat elements that are important in early developmental stages and in disease modeling among primate species [31]. Furthermore, we have investigated some genes with significant roles in PSC biology. The tree topology of *KLF4* and *c-Myc*, 2 important genes in PSC biology [32], also followed the general consensus trees (Fig. 4A). PSCl genes are of particular interest because of adaptation of pig-tailed macaque iPSCs in maintaining pluripotency in human pluripotent culture media [19]. We also observed that human Wnt family member 8A (*WNT8A*) transcript variant 1 (NM_0 013 00938) has an orthologous transcript in pig-tailed macaques but lacks an orthologous transcript in rhesus or cynomolgus macaques.

The presented assembly is of a single individual, while the human genome assembly increasingly represents some of the variations in highly variable regions across many individuals. There is a need for enhancing this assembly with a representation of pig-tailed macaque populations in the future.

## Conclusions

The chromosome-level assembly of the pig-tailed macaque genome provides a useful resource for the study of genome rearrangements, structural variation, and their possible roles in disease susceptibility among primates. In addition, the gene orthology relationship and reconstruction of phylogenetic trees among the 3 macaque species and human provide new insights into their evolutionary history. We investigated the evolutionary distance among the 3 macaque species and human with regards to the IM and PSC genes. Further investigation of evolutionary distances among these primate species and humans based on these and other gene sets can facilitate the selection of appropriate animal models for the study of different human diseases.

## Materials and Methods

### Study subject, sample preparation, and DNA extraction

The study subject is a 14-year-old male pig-tailed macaque (*M. nemestrina*) from the pig-tailed macaque colony at Johns Hopkins University. A blood sample was obtained from the study animal following the Institutional Animal Care and Use Committee protocol at Johns Hopkins University. Peripheral blood mononuclear cells (PBMCs) were isolated from whole blood collected in acid citrate dextrose from the male pig-tailed macaque Ma2 by percoll gradient centrifugation. After separation, cells were stored viably in 90% fetal bovine serum/10% dimethyl sulfoxide in liquid nitrogen [33]. Approximately 1,000,000 PBMCs were used for the extraction of high molecular weight (HMW) DNA using the MagAtrract HMW DNA Kit (Qiagen, Inc., Valencia, CA, USA). The average size of the HMW DNA extracted DNA, ~53 kb (Supplementary Fig. S1B), was measured using a Fragment Analyzer (Advanced Analytical Technologies, Inc., Ankeny, IA, USA). Additionally, iPSCs were derived from PBMCs of the study animal [19].

### Chromosome karyotyping

The macaque-derived (*M. nemestrina*) iPSC line derived from the study animal's PBMCs was harvested by standard cytogenetic methodology of mitotic arrest, hypotonic shock, and fixation with 3:1 methanol–acetic acid. Chromosome slide preparations were stained by G-banding and classified by the standard *M. nemestrina* karyotype 1, 2. Analysis of 20 metaphase cells demonstrated an apparently normal male karyotype of 20 autosomes and 2 sex chromosomes (X and Y) in all cells [14, 34].

### Library preparation and sequencing

To generate a chromosome-level assembly, we prepared 2 different genome libraries.

#### Linked-read library preparation

The 10X Genomics Genome Chromium platform (10X Genomics, Pleasanton, CA, USA) including Gel Bead-In-EMulsions (GEMs) generation and barcoding was used to generate the linked-read library [35]. In summary, in a 10X Genomics Chromium microfluidic Genome Chip ~0.8–1.2 ng of HMW DNA was combined with master mix and partitioning oil to create GEMs. Single HMW DNA molecules in each Gel Bead were barcoded with a unique 10X Genomics barcoded primer. Upon dissolution of the Genome Gel Bead in the GEM, primers containing (i) an Illumina® R1 sequence (Read 1 sequencing primer), (ii) a 16-nucleotide 10X Genomics Barcode, and (iii) a 6-nucleotide random primer sequence were added into the fragments of DNA. The barcoded fragments of DNA ranging from a few to several hundred base pairs were pooled together when the GEMs were broken after incubation. Silane magnetic beads were used for the post GEM generation cleanup to remove leftover biochemical reagents. Solid Phase Reversible Immobilization (SPRI) beads were used to optimize the appropriate DNA size range for library preparation (AMPure XP, BeckmanCoulter, Inc, Indianapolis, IN, USA). Read 1 sequence and 10X Genomic barcodes were added during the GEM generation, and P5, P7 primers, Read 2, and sample index were added during library construction after end repair, A-tailing, and adaptor ligation and amplification. The constructed library was evaluated on a Bioanalyzer 2100 using Agilent High Sensitivity DNA (Agilent, Inc., Santa Clara, CA, USA) and quantified using Kapa Library Quantification Kit (KappaBiosystems, Inc., Wilmington, MA, USA). The sequencing of each sample was conducted on 3 lanes on Illumina HiSeq 4000 (Illumina HiSeq 4000, RRID:SCR_016386) sequencing platform that generated ~1.8 billion of paired-end read sequencing data with a 2 × 151 bp read size (R1 and R2).

#### HiC library preparation by Dovetail Genomics

3 Dovetail HiC libraries were prepared as described previously [13]. Briefly, for each library, chromatin was fixed in place with formaldehyde in the nucleus and then extracted. Fixed chromatin was digested with DpnII, the 5′ overhangs filled in with biotinylated nucleotides, and then free blunt ends were ligated. After ligation, crosslinks were reversed and the DNA purified from protein. Purified DNA was treated to remove biotin that was not internal to ligated fragments. The DNA was then sheared to a ~350 bp mean fragment size, and sequencing libraries were generated using NEBNext Ultra enzymes and Illumina-compatible adapters. Biotin-containing fragments were isolated using streptavidin beads before PCR enrichment of each library. The libraries were sequenced on an Illumina Hi-seq 4000 sequencing platform (2 × 151 bp) (Illumina Hi-seq 4000, RRID:SCR_016386). The number and length of read pairs produced for each library were 141 million for library 1, 88 million for library 2, and 133 million for library 3. Together, these Dovetail HiC library reads provided 1990.23× physical coverage of the genome (1–50 kb pairs).

### Genome assembly

Next, we assembled and scaffolded the data. The genome assembly was carried out using 10X Genomics' Supernova 1.2.0 (10X Genomics Inc, Pleasanton, CA, USA) with default parameters, and the resulting assembly scaffolded using Dovetail Genomics' HiRise scaffolder.

#### Linked-read library

First, we assembled the linked reads using Supernova 1.2.0 (10X Genomics, Inc, Pleasanton, CA, USA) with default parameters on the Amazon Web Services (AWS) x1.16xlarge instance with 976 GB computing memory and 2 Tb of SSD disk and i3.16xlarge instance with 488 GB computing memory and 2 TB of NVMe disk. The genome assembly of an organism with a diploid genome size close to that of the human genome (~3.2 Gb) requires ~284 GB of memory and 2 TB of disk space for the data storage. The assembly quality was assessed using BUSCO 3.0.2 (BUSCO, RRID:SCR_015008) [16] (Supplementary Fig. S1A).

#### Scaffolding the assembly using HiRise

The input *de novo* assembly, shotgun reads, and Dovetail HiC library reads were used as input data for HiRise, a software pipeline designed specifically for using proximity ligation data to scaffold genome assemblies [36]. Shotgun and Dovetail HiC library sequences were aligned to the draft input assembly using a modified SNAP 1.0beta.16 read mapper [37]. The separations of HiC read pairs mapped within draft scaffolds were analyzed by HiRise to produce a likelihood model for genomic distance between read pairs, and the model was used to identify and break putative misjoins, to score prospective joins, and make joins above a threshold. After scaffolding, shotgun sequences were used to close gaps between contigs. As pointed out in previous studies [38], topological domains throughout the genome can show non-random associations, which can potentially cause issues during the scaffolding process. However, these issues should be minor in the presented genome architecture because the baseline genome assembly showed a high scaffold N50 and thus likely had a continuity larger-than-TAD scale. Another common issue with HiC scaffolding is the lower amount of links present for short scaffolds, which can lead to wrongly inverted scaffolds. We cannot rule out that our genome assembly still includes a few of these smaller inversions.

### Repeat identification

We identified repeats in the genome using a combination of *de novo* repeat finding with RepeatModeler v1.08 (RepeatModeler, RRID:SCR_015027) and homology-based repeat annotation using RepeatMasker 4.0.8 (RepeatMasker, RRID:SCR_012954) and the RepBase Dfam 3.0 database. For the latter we applied the gccalc option in addition to the default parameters to calculate GC content separately for each contig/scaffold. These repeats include simple repeats (1–5 bases), tandem repeats, duplication of 100–200 bases that are more typically found at centromeres and telomeres of chromosomes, segmental duplications (large blocks of 10–300 kb), processed pseudogenes, retrotranscripts, SINES, LINES, DNA transposons, and retrovirus retrotransposons.

### Synteny analysis

To conduct synteny analysis between pig-tailed macaques and humans and between pig-tailed and rhesus macaques, we first carried out pairwise whole-genome alignments using Satsuma Synteny 3.1.0 [39] with default parameters. Next, we plotted the links using Circos 0.69.6 [40].

### Chromosome assignment to the 23 largest scaffolds

We took 2 approaches to assign scaffolds to chromosomes: (i) We used synteny analysis between pig-tailed and rhesus macaque to identify chromosomes of rhesus macaque genome that align to each of the 23 largest pig-tailed macaque scaffolds. (ii) We blasted [41] validated genes with known loci on rhesus macaque genome onto the pig-tailed macaque genome to verify the assignments using the synteny information.

### Genome annotation

RNAseq: messenger RNA was extracted from the iPSCs of the pig-tailed macaque using RNeasy Mini Kit extraction kit (Qiagen, Inc., Valencia, CA, USA). Following complementary DNA synthesis, the RNAseq library was prepared using an Illumina TrueSeq RNA Library Prep kit V2 (Illumina, Inc., San Diego, CA, USA) with unique dual index to avoid index switching [42]. Sequencing was conducted using a HiSeq4000 Illumina machine generating ~50 million paired reads (2 × 150 bp, Illumina, Inc.). The RNAseq data were subsequently used for annotation steps.

Gene annotation was then conducted using the software Maker 2.31.9 (MAKER, RRID:SCR_005309) [27]. For the annotation of the genome we used RNAseq and proteomics data from the study animal's PBMCs and iPSCs (generated from the PBMCs). To do so, RNAseq data were aligned to the genome using HiSat 2.1.0 [43], and the assembly was conducted using StringTie 1.3.3 (StringTie, RRID:SCR_016323) [44]. In addition to the RNAseq data from iPSC of the study animal, we added the pig-tailed macaque RNAseq and proteomics data available on the Ensembl and NCBI databases, as well as the proteomics data from the human Genecode database.

### Proteomics sample preparation

Cell pellets were lysed in 100 μL 6 M guanidinium chloride, 10 mM tris(2-carboxyethyl) phosphine (TCEP), 40 mM chloroacetamide (CAA), and 100 mM tris(hydroxymethyl)aminomethane (Tris) pH 8.5 buffer. Lysates were incubated at 95°C for 5 min and briefly sonicated. Samples were boiled for 5 min at 95°C and vortexed every 1 min, then spun 5 min + 10 min + 10 min at 12,000*g*, taking supernatant for each step to continue. Protein concentration was measured using the BCA method. Trypsin (modified, from Promega, Madison, WI, USA) was added at a protein to enzyme ratio of 50:1. Samples were incubated overnight at 37°C. Peptides were added to trifluoroacetic acid to 1% then cleaned up using an Oasis HLB cartridge (1 mm^3^/10 mg, Waters). Samples were dried by means of speed-vacuum drying method and dissolved in 100 mM Triethylamonium bicarbonat (TEAB). Peptides were labeled with TMT 10plex reagent (Thermo Fisher) and combined at equal amounts, then vacuum spun until dry.

Waters 2D liquid chromatography (Waters MClass 2DnLC) was used for peptide separation by reverse phase chromatography at high pH in the first dimension followed by an orthogonal separation at low pH in the second dimension. In the first dimension, the mobile phases were buffer A (20 mM ammonium formate at pH 10) and buffer B (acetonitrile). Peptides were separated on an Xbridge 300 μm x 5 cm C18 5.0 μm column (Waters) using 15 discontinuous step gradients at 2 μL/min. In the second dimension, peptides were loaded to an in-house packed 100 μm ID/15 μm tip ID × 28 cm C18-AQ 1.8 μm resin column with buffer A (0.1% formic acid in water). Peptides were separated with a linear gradient from 5% to 40% buffer B (0.1% formic acid in acetonitrile) at a flow rate of 300 nL/min in 112, 170, and 180 min. The LC system was directly coupled in-line with an Orbitrap Fusion Lumos Tribrid Mass Spectrometer (Thermo Fisher Scientific) via a Thermo nanoelectrospray source. The machine was operated at 1.8–2.2 kV to optimize the nanospray with the ion transfer tube at 275°C. The mass spectrometer was run in a data-dependent mode. The full MS scan was acquired in the Orbitrap mass analyzer with resolution 120,000 at *m*/*z* 375–1,500 followed by top 20 tandem mass spectroscopy in ion trap. For all sequencing events dynamic exclusion was enabled to fragment peptide once and excluded for 60 s.

### Proteomics data processing and analysis

The raw data were then processed with the software Proteome Discoverer (Proteome Discoverer, RRID:SCR_014477) (Thermofisher Scientific, Inc.). Mass tolerance of 10 ppm was used for precursor ion and 0.6 Da for fragment ions for the database search. The search included cysteine carbamidomethylation as a fixed modification. Acetylation at protein N-terminus and methionine oxidation were used as variable modifications. Up to 2 missed cleavages were allowed for trypsin digestion. Only unique peptides with a minimum length of 6 amino acids were considered for protein identification. The FDR was set as <1%.

### Maker Proteomics for gene identification

We conducted a 2-step search. The first search was against our Ma2 annotated database; the unmatched spectra were searched against the Human Gencode19 (GENCODE, RRID:SCR_014966). Our Ma2 annotation database identified 9,031 proteins, and the human database identified an additional 4,531 proteins for a total of 13,562 identified proteins with FDR 1%.

In a further attempt to improve the annotation using Maker 2.31.9 (MAKER, RRID:SCR_005309) [27], we added the GENCODE 28 database [26] to the homology-based annotation step in order to increase the number of annotated genes on the pig-tailed macaque genome. After adding the GENCODE genes to a new Maker run, the total number increased to 13,797 master proteins. This included 3,979 proteins from the GENCODE human database and 9,818 proteins from the initial pig-tailed macaque Ma2 annotation. The Thermo Proteome Discoverer 2.1.0.81 software (Proteome Discoverer, RRID:SCR_014477) was used for protein mapping.

### Pairwise bit score comparison

We applied POFF (proteinortho5.pl) [28] using default parameters to find orthologous sequences between the 3 macaque species and human, using mouse as an outgroup.

From the POFF (proteinortho5.pl) output, we extracted the bit scores (a statistical value that represents sequence similarity) of each gene for their distance from human to each of the macaque species. We aggregated the bit scores for each gene in the gene sets of interest (list of innate immune genes and the list of pluripotent stem cell genes) by computing the mean over corresponding orthologs.

### Reconstruction of phylogenetic relationships between the 3 macaque species and human

The POFF software (proteinortho5.pl) [28] with optimized parameters (-p = blastn+ -project = Ma2 -graph -singles-p = blastn+ -project = Ma2 -graph -singles) was used to detect orthology between gene sequences from the 5 species, pig-tailed macaque, rhesus macaque (*M. mulatta*8.0.1), cynomolgus macaque (*M. fascicularis*5.0), human (GRCh38/hg38), and mouse (GRCm38/mm10) [45]. The pig-tailed macaque sequences were derived from our own assembly and annotation of the genome. The mouse sequences were included for comparison and as an outgroup for rooting the subsequently estimated evolutionary trees.

In the next step, MAFFT 7.310 (MAFFT, RRID:SCR_011811) [46, 47] was applied to individual ortholog groups, each containing 5 sequences corresponding to the species listed above. Aligned sequences were subsequently used as inputs for RAxML 8.2.10 (RAxML, RRID:SCR_006086) [29] with optimized parameters (raxmlHPC -f a -x 12 345 -p 12 345 -N 100 -m GTRGAMMA -s -n) to reconstruct the phylogenetic relationships between the 5 species. The Astral 5.6.2 program [30] with default settings was then applied to generate a species tree from the individual gene trees. Next, the gene trees were rooted using mouse as outgroups. We reconstructed a total of 15 possible phylogenetic tree topologies. We explored the assignment of all genes as well as the subset of immune genes (IM) and PSC genes to the 15 tree topologies. This can provide information on which species are closer to or farther from human.

## Availability of Supporting Data and Materials

All supporting data and materials are available in the *GigaScience* database, GigaDB [48].

## Additional Files

**Supplementary Figure S1**. (A) BUSCO 3.0.2 for assembly evaluation. (B) Extracted HMW DNA average size and molecular size distribution.

**Supplementary Figure S2**. Venn diagram of the number of common orthologs (A) among rhesus macaque (RM), pig-tailed macaque (PM), and human and (B) among cynomolgus macaque (CM), RM, PM, and human.

**Supplementary Figure S3**. Mean bit score ortholog difference to the respective corresponding human ortholog for (A) innate immune genes (IM) between pig-tailed macaque (PM) and cynomolgus macaque (CM) (left), between CM and rhesus macaque (middle), and between PM and rhesus macaque (right). The bit score differences for most of the genes are almost equal to zero. Rhesus and cynomolgus macaques are indicated by data points clustering along the 45° line. (B) Pluripotent stem cell genes (PSC) between PM and CM (left), between CM and rhesus macaque (middle), and between PM and rhesus macaque (right). The bit score differences for most of the genes are almost equal to zero. Rhesus and cynomolgus macaques exhibit data points clustering along the 45° line.

**Supplementary Figure S4**. Possible phylogenetic tree topologies among 3 macaque species and human, using mouse as an outgroup. Tree 1 is supported by the majority of the conserved genes among the 5 species, 52.8% (2,339/4,434). Tree 6 and Tree 10 indicate a closer relationship of pig-tailed macaque to human than either of the other 2 macaque species. There were 22 genes that follow Tree 6 topology and 13 genes that follow Tree 10 topology.

giaa069_GIGA-D-19-00333_Original_Submission

giaa069_GIGA-D-19-00333_Revision_1

giaa069_Response_to_Reviewer_Comments_Original_Submission

giaa069_Reviewer_1_Report_Original_Submission Xiao-Guang Qi -- 10/25/2019 Reviewed

giaa069_Reviewer_2_Report_Original_Submission Taras K Oleksyk, Ph.D. -- 12/3/2019 Reviewed

giaa069_Supplemental_Files

## Abbreviations

BLAST: Basic Local Alignment Search Tool; bp: base pairs; BUSCO: Benchmarking Universal Single-Copy Orthologs; CM: cynomolgus macaque; FDR: false discovery rate; Gb: gigabase pairs; GC: guanine-cytosine; HMW: high molecular weight; IM: innate immune; iPSC: induced pluripotent stem cell; kb: kilobase pairs; LINE: long interspersed nuclear element; LTR: long terminal repeat; Mb: megabase pairs; ML: maximum likelihood; NCBI: National Center for Biotechnology Information; NHP: nonhuman primates; NIH: National Institutes of Health; PM: pig-tailed macaque; PBMC: peripheral blood mononuclear cell; PSC: pluripotent stem cell genes; RAxML: Randomized Axelerated Maximum Likelihood; RM: rhesus macaque; RNAseq: RNA sequencing; SINE: short interspersed nuclear element; TE: transposable element.

## Competing Interests

The authors declare that they have no competing interests.

## Funding

This work used the Genome Sequencing Service Center by Stanford Center for Genomics and Personalized Medicine Sequencing Center, supported by the NIH grant award S10OD020141, Stanford Center for Personal Dynamics Regulomes supported by the NIH grant award PARYB (SPO-112285), and samples from the Johns Hopkins Pigtailed Macaque Breeding Colony supported by NIH U42OD013117.

## Authors' Contributions

M.R., M.P.S., D.G.S., and J.L.M. conceived the study. M.R. designed and conducted experimental aspects of assembly using linked-read and HiC methods. M.R. and M.A. performed high molecular weight DNA extraction and linked-read library preperation. M.R. and Hayan Lee conducted assembly pipeline. M.R. and A.B. conducted repeat annotation. Hoyong Lee and G.S. conducted BUSCO analysis. M.R., A.B., and W.Z. conducted synteny analysis. M.R. and R.S. conducted RNAseq analysis and RNAseq assembly. L.J. and R.J. performed proteomics experiments and analysis. M.R., A.B., and J.B.H. conducted genome annotation. M.R., L.H.N., A.B., and S.P. conducted cross-species orthologous comparison and evolutionary tree reconstruction. H.C. supervised and monitored library sequencing quality. S.H. supervised statistical methods for cross-species ortholog and tree analysis, S.B. supervised assembly approach, and I.L.W. and J.L.M. supervised immunology stem cell biology genes tree analysis. D.G.S. supervised evolutionary tree analysis. S.P. guided all the analysis steps conducted in the study. M.P.S. supervised the study.
