# Supplementary material for: Chromosome-level de novo assembly of the pig-tailed macaque genome using linked-read sequencing and HiC proximity scaffolding
Source: Gigascience. 2020 Jul 10;9(7):giaa069. doi: 10.1093/gigascience/giaa069 (PMC7350979; doi:10.1093/gigascience/giaa069)
Supplement: giaa069_Response_to_Reviewer_Comments_Original_Submission [file giaa069_response_to_reviewer_comments_original_submission.pdf]

Author's Response To Reviewer Comments

Close

We highly appreciate the constructive feedback from the reviewers of the manuscript. According to the reviewers' comments, we made changes in the manuscript and addressed the comments of the reviewers which helped us improve the quality of this manuscript. Please see below the changes we made in the manuscript according to the reviewers' comments.
Thank you again for the helpful feedback. Please see below the changes.

Reviewer reports:

Reviewer #1: The authors present an assembly of pig-tailed macaque with the combination of long read sequencing and Hi-C. The gene annotation is well done using transcriptome and proteomics data. Overall, the manuscript is clear to follow. Some questions may be helpful to improve the manuscript.

Both package versions and parameter settings of the software used should be given.

Gene names should be italic.

The manuscript would benefit from some comparison of how much better the genome assembly is relative to previous assembly. For example, a comparative repeat analysis, or what specific regions of the assembly were absent in the previous version, what do they contain, how many gaps were filled.

This combination method increased the scaffold N50 largely, however, the statics at the contig level were not reported.

Page 14. The sentence "Here we present, to our knowledge, the first linked-reads and HiC- based assembly of a primate genome." in the discussion section may be not right. In my knowledge, these technologies have been used in the genome assembly of Rhinopithecus roxellana, a golden sub nosed monkey.

The version of genome assembly for other species used for genome comparison should be detailed.

The legend for figure 3 is not adequate. What is the reader supposed to conclude from the figure?

Reviewer #2: The paper used an HiC approach to capture the three dimensional structure of the chromosomes inside the cell, leading to much more contiguous reconstruction of the genome that was previously possible. The resulting pig-tailed macaque genome covers 90% of the combined chromosome length, and is similar to the human genome int its scaffold size length. Specifically, using combination of linked reads and proximity ligation, the authors were able to increase scaffold N50 to 127.5 Mb and then assigned to chromosomes and annotated with resequenced mRNA from IPSC cells. This paper represents a significant improvement over the past assemblies of the macaque genome and is a very needed contribution in the field of primate comparative genomics.

I only have several minor comments.

1. It would be good for the authors to explain how they have addressed the main problem with the HiC data: 1) nonrandom association between topological domains (described in Dixon et al., 2012) and 2) the problem with accurate orientation of the inversions within the scaffolds.
2.The authors assume that macaque repeats are identical to humans, and run Repeat Masker. It is at least theoretically possible that there are unknown types of repeats in this species. I would therefore recommend to run Repeat Modeller as well to account for this possibility

Changes according to reviewers' comments:

Reviewer #1:

1. The package version and parameter setting of the software used were added into the method section where each software was mentioned. We also attach below the list and the version of all software used in this study. We mentioned the parameters of software and database in the method section. We used default parameter settings for most of the software used. The details of slights changes in parameter settings have been mentioned in the method section.
a. Supernova /1.2.0
b. BUSCO /3.0.2
c. Satsuma Synteny 3.1.0
d. Circos 0.69.6
e. SNAP /1.0beta.16
f. RepeatMasker/4.0.8
g. Maker /2.31.9
h. HiSat /2.1.0
i. POFF(proteinortho5.pl)
j. RAxML/8.2.10
k. MAFFT/7.310
l. Astral 5.6.2
m. Dfam 3.0
2. The gene names were changed to italic format.
3. We conducted RepeatModeler to identify the new repeats that we identified based on this chromosome level assembly and these repeats were not previously identified or listed in previous assembly or RepBase database. We found additional 1.28% repeats specific to this species that was not identified before. The results of the RepeatModeler are attached to this resubmission of the paper.
4. Also, we attach to this resubmission a detailed statistics of the assemblies either with Linkedread or the combination of linked-read assembly and HiC method (Linkedread + HiC).
5. We deleted the sentence "Here we present, to our knowledge, the first linked-reads and HiC- based assembly of a primate genome." on Page 14 from the discussion.
6. We added the version of the genome assembly of the other species used for the analysis.
7. We added more details to the legend of figure 3.

Reviewer #2:

1. We used Dovetail's HiRise pipeline for the scaffolding, and we have asked them to help with the clarification of the HiC method for assembly as explained below:

"1.HiRise does not directly address issues of distinguishing proximity ligation associations due to topological domains from those due to the correlation between proximity and linear separation on a DNA molecule. When run with Hi-C data only, this issue is minimized by having the input assembly contiguity at a larger-than-TAD scale, say contig N50 of 500 kbp to 1 Mbp and up

2. Hi-C data does not in general provide enough resolution to avoid inversion errors for smaller contigs, since in each possible orientation, the Hi-C data have similar numbers of links. For larger contigs, > 500kbp, Hi-C links for the correct orientation dominate and scaffolds are correct. This is a property of Hi-C data independent of scaffolding tool and not due to TADs in particular."
We have added a short paragraph about that into the Materials and Methods section.

2. We conducted RepeatModeler to identify the new repeats that we identified based on this chromosome level assembly and these repeats were not previously identified or listed in previous assembly or RepBase database. We found additional 1.28% repeats specific to this species that was not identified before. The results of the RepeatModeler are attached to this resubmission of the paper.

Close
