# Supplementary material for: Chromosome-level de novo assembly of the pig-tailed macaque genome using linked-read sequencing and HiC proximity scaffolding
Source: Gigascience. 2020 Jul 10;9(7):giaa069. doi: 10.1093/gigascience/giaa069 (PMC7350979; doi:10.1093/gigascience/giaa069)
Supplement: giaa069_Reviewer_1_Report_Original_Submission — Xiao-Guang Qi -- 10/25/2019 Reviewed [file giaa069_reviewer_1_report_original_submission.pdf]

Reviewer Report

Title: Chromosome-level de novo assembly of the pig-tailed macaque genome using linked-read sequencing and HiC proximity scaffolding.

Version: Original Submission **Date:** 10/25/2019

Reviewer name: Xiao-Guang Qi

Reviewer Comments to Author:

The authors present an assembly of pig-tailed macaque with the combination of long read sequencing and Hi-C. The gene annotation is well done using transcriptome and proteomics data. Overall, the manuscript is clear to follow. Some questions may be helpful to improve the manuscript.
Both package versions and parameter settings of the software used should be given.
Gene names should be italic.
The manuscript would benefit from some comparison of how much better the genome assembly is relative to previous assembly. For example, a comparative repeat analysis, or what specific regions of the assembly were absent in the previous version, what do they contain, how many gaps were filled.
This combination method increased the scaffold N50 largely, however, the statics at the contig level were not reported.
Page 14. The sentence "Here we present, to our knowledge, the first linked-reads and HiC- based assembly of a primate genome." in the discussion section may be not right. In my knowledge, these technologies have been used in the genome assembly of Rhinopithecus roxellana, a golden sub nosed monkey.
The version of genome assembly for other species used for genome comparison should be detailed.
The legend for figure 3 is not adequate. What is the reader supposed to conclude from the figure?

Level of Interest

Please indicate how interesting you found the manuscript: Choose an item.

Quality of Written English

Please indicate the quality of language in the manuscript: Choose an item.

Declaration of Competing Interests

Please complete a declaration of competing interests, considering the following questions:

- Have you in the past five years received reimbursements, fees, funding, or salary from an organisation that may in any way gain or lose financially from the publication of this manuscript, either now or in the future?

- Do you hold any stocks or shares in an organisation that may in any way gain or lose financially from the publication of this manuscript, either now or in the future?
- Do you hold or are you currently applying for any patents relating to the content of the manuscript?
- Have you received reimbursements, fees, funding, or salary from an organization that holds or has applied for patents relating to the content of the manuscript?
- Do you have any other financial competing interests?
- Do you have any non-financial competing interests in relation to this paper?

If you can answer no to all of the above, write 'I declare that I have no competing interests' below. If your reply is yes to any, please give details below.

I declare that I have no competing interests

I agree to the open peer review policy of the journal. I understand that my name will be included on my report to the authors and, if the manuscript is accepted for publication, my named report including any attachments I upload will be posted on the website along with the authors' responses. I agree for my report to be made available under an Open Access Creative Commons CC-BY license (http://creativecommons.org/licenses/by/4.0/). I understand that any comments which I do not wish to be included in my named report can be included as confidential comments to the editors, which will not be published.

Choose an item.

To further support our reviewers, we have joined with Publons, where you can gain additional credit to further highlight your hard work (see: https://publons.com/journal/530/gigascience). On publication of this paper, your review will be automatically added to Publons, you can then choose whether or not to claim your Publons credit. I understand this statement.

Yes Choose an item.
