# Supplementary material for: Chromosome-level de novo assembly of the pig-tailed macaque genome using linked-read sequencing and HiC proximity scaffolding
Source: Gigascience. 2020 Jul 10;9(7):giaa069. doi: 10.1093/gigascience/giaa069 (PMC7350979; doi:10.1093/gigascience/giaa069)
Supplement: giaa069_Reviewer_2_Report_Original_Submission — Taras K Oleksyk, Ph.D. -- 12/3/2019 Reviewed [file giaa069_reviewer_2_report_original_submission.pdf]

Reviewer Report

Title: Chromosome-level de novo assembly of the pig-tailed macaque genome using linked-read sequencing and HiC proximity scaffolding.

Version: Original Submission **Date:** 12/3/2019

Reviewer name: Taras K Oleksyk, Ph.D.

Reviewer Comments to Author:

The paper used an HiC approach to capture the three dimensional structure of the chromosomes inside the cell, leading to much more contiguous reconstruction of the genome that was previously possible. The resulting pig-tailed macaque genome covers 90% of the combined chromosome length, and is similar to the human genome int its scaffold size length. Specifically, using combination of linked reads and proximity ligation, the authors were able to increase scaffold N50 to 127.5 Mb and then assigned to chromosomes and annotated with resequenced mRNA from IPSC cells. This paper represents a significant improvement over the past assemblies of the macaque genome and is a very needed contribution in the field of primate comparative genomics.
I only have several minor comments.
1. It would be good for the authors to explain how they have addressed the main problem with the HiC data: 1) nonrandom association between topological domains (described in Dixon et al., 2012) and 2) the problem with accurate orientation of the inversions within the scaffolds.
2.The authors assume that macaque repeats are identical to humans, and run Repeat Masker. It is at least theoretically possible that there are unknown types of repeats in this species. I would therefore recommend to run Repeat Modeller as well to account for this possibility

Level of Interest

Please indicate how interesting you found the manuscript: Choose an item.

Quality of Written English

Please indicate the quality of language in the manuscript: Choose an item.

Declaration of Competing Interests

Please complete a declaration of competing interests, considering the following questions:

- Have you in the past five years received reimbursements, fees, funding, or salary from an organisation that may in any way gain or lose financially from the publication of this manuscript, either now or in the future?
- Do you hold any stocks or shares in an organisation that may in any way gain or lose financially from the publication of this manuscript, either now or in the future?

- Do you hold or are you currently applying for any patents relating to the content of the manuscript?
- Have you received reimbursements, fees, funding, or salary from an organization that holds or has applied for patents relating to the content of the manuscript?
- Do you have any other financial competing interests?
- Do you have any non-financial competing interests in relation to this paper?

If you can answer no to all of the above, write 'I declare that I have no competing interests' below. If your reply is yes to any, please give details below.

I have no competing interests

I agree to the open peer review policy of the journal. I understand that my name will be included on my report to the authors and, if the manuscript is accepted for publication, my named report including any attachments I upload will be posted on the website along with the authors' responses. I agree for my report to be made available under an Open Access Creative Commons CC-BY license (http://creativecommons.org/licenses/by/4.0/). I understand that any comments which I do not wish to be included in my named report can be included as confidential comments to the editors, which will not be published.

Choose an item.

To further support our reviewers, we have joined with Publons, where you can gain additional credit to further highlight your hard work (see: https://publons.com/journal/530/gigascience). On publication of this paper, your review will be automatically added to Publons, you can then choose whether or not to claim your Publons credit. I understand this statement.

Yes Choose an item.
